# Inhibition Effect of Triglyceride Accumulation by Large Yellow Croaker Roe DHA-PC in HepG2 Cells

**DOI:** 10.3390/md17090485

**Published:** 2019-08-21

**Authors:** Xiaodan Lu, Rongbin Zhong, He Sun, Baodong Zheng, Lijiao Chen, Song Miao, Peng Liang

**Affiliations:** 1 College of Food Science, Fujian Agriculture and Forestry University, Fuzhou 350002, China; 2 China-Ireland International Cooperation Centre for Food Material Science and Structure Design, Fujian Agriculture and Forestry University, Fuzhou 350002, China; 3 Fujian Provincial Key Laboratory of Quality Science and Processing Technology in Special Starch, Fujian Agriculture and Forestry University, Fuzhou 350002, China; 4 Food Chemistry and Technology Department, Teagasc Food Research Centre, Moorepark, Fermoy, P61 C996 Co. Cork, Ireland; 5 Key Laboratory of Marine Biotechnology of Fujian Province, Institute of Oceanology, Fujian Agriculture and Forestry University, Fuzhou 350002, China

**Keywords:** large yellow croaker roe, DHA-PC, hyperlipidemia, regulatory effect, mechanism

## Abstract

The phospholipids (PLs) of large yellow croaker (*Pseudosciaena crocea*, *P. crocea*) roe contain a high level of polyunsaturated fatty acids, especially docosahexaenoic acid (DHA), which can lower blood lipid levels. In previous research, PLs of *P. crocea roe* were found able to regulate the accumulation of triglycerides. However, none of these involve the function of DHA-containing phosphatidylcholine (DHA-PC), which is the main component of PLs derived from *P. crocea* roe. The function by which DHA-PC from *P. crocea* roe exerts its effects has not yet been clarified. Herein, we used purified DHA-PC and oleic acid (OA) induced HepG2 cells to establish a high-fat model, and the cell activity and intracellular lipid levels were then measured. The mRNA and protein expression of Fatty Acid Synthase (FAS), Carnitine Palmitoyl Transferase 1A (CPT1A) and Peroxisome Proliferator-Activated Receptor α (PPARα) in HepG2 cells were detected via RT-qPCR and western blot as well. It was found that DHA-PC can significantly regulate triglyceride accumulation in HepG2 cells, the effect of which was related to the activation of PPARα receptor activity, upregulation of CPT1A, and downregulation of FAS expression. These results can improve the understanding of the biofunction of hyperlipidemia mediated by DHA-PC from *P. crocea* roe, as well as provide a theoretical basis for the utilization of DHA-PC from *P. crocea* roe as a functional food additive.

## 1. Introduction

Fish roe has a high percentage of phosphatidylcholine (PC) [1,2], which has been shown to be effective for the improvement of learning ability and the reduction of plasma lipids [3,4]. Fish roe has also been reported to contain large amounts of n-3 polyunsaturated fatty acids (n-3 PUFAs), mainly eico-sapentaenoic acid (EPA) and docosahexaenoic acid (DHA), which have been determined to be able to prevent coronary heart disease, cardiovascular diseases [5], inflammation [6], cancer [7] and so on. However, the applicability of bioactive compounds can be limited by their low stability, which leads to their consequent degradation and loss of activity. Liposomes have attracted wide attention due to their high bioavailability, biodegradability, safety, and amphiphilicity. They have considerable potential for application in the food industry to encapsulate and protect nutraceuticals and other bioactive agents, such as vitamins, polyphenols, enzymes, carotenoids, and fatty acids [8,9,10,11]. At present, liposomes have been widely used as drug carriers [12,13,14,15]. Although liposomes have been used for medical and cosmetic purposes, their applications in food systems are relatively new [16].

Recently, these PLs containing polyunsaturated fatty acids (PUFAs) have received much attention because of their health benefits. Some reports have shown that unsaturated PLs provide numerous advantages in the prevention and treatment of cardiovascular disease, autoimmune disorders and neurological disease [17,18]. Marine PLs are much high in PUFAs [19]. Tsoupras et al. reported that several marine sources contain bioactive PLs, which has strong antithrombotic and atherosclerotic cardioprotective activities [20]. Nasopoulou et al. found that fish PLs retard atherosclerosis by upregulating platelet-activating factor (PAF) catabolism and down-regulating PAF biosynthesis in rabbits [21]. Most n-3 PUFAs from fish roe are present in the phospholipid (PL) form, of which PC is the predominant lipid class [1,22]. PLs containing n-3 PUFAs possess stronger bioactivities than the n-3 PUFAs. Rossmeisl et al. found that PLs rich in n-3 PUFAs can prevent non-alcoholic fatty liver disease [23]. PC, DHA, and EPA have many unique biological activities based on their own functional properties. Some studies have reported that their combination could have more powerful effects on adjusting liver and blood plasma lipid levels [24,25]. There is evidence that PLs combined with n-3 PUFAs (such as EPA and DHA) are more efficiently incorporated into tissue membranes [19]. Previous reports have also demonstrated that fish roe contained a high amount of DHA-containing phospholipid (DHA-PL) [26,27].

*P. crocea* is known among consumers for its delicious taste and high nutritional value [28,29]. Especially in the southern part of China, people are accustomed to eating *P. crocea* and its roe, and the utilization of *P. crocea* roe has attracted the attention of many researchers [30]. We have found that *P. crocea* roe contained high levels of total lipids (19.6 ± 1.32%, w/w) and phospholipids (61.2 ± 1.22% of the total lipid, w/w) [31]. Roe is a major byproduct of *P. crocea* processing and is a potential source of DHA-PL, which have a high amount of DHA (31.0 ± 0.19% of the total phospholipids, w/w) and EPA as well as high content of PC (61.06 ± 0.02% of the total phospholipids, w/w) and Phosphatidylethanolamine (PE) [31]. Further study demonstrated that DHA-PL was able to prevent fatty degeneration of liver tissues and reduce the risk of atherosclerosis [32,33]. However, there are few reports on the effects of DHA-PC, and the mechanisms by which it acts have not yet been elucidated.

Because of the large size of the *P. crocea* roe, and in particular its PLs, has more potential to be exploited. Furthermore, considering the high proportion of PCs in PLs from *P. crocea* roe, marine PCs are worthy of further exploitation. In this study, we explored the effect of DHA-PC from *P. crocea* roe on triglyceride accumulation in HepG2 cells, which was done for the first time by establishing the high-fat cell model. We also used RT-qPCR and western blot to initially illuminate the mechanism by which the DHA-PC exerts these effects. These results would enhance our understanding of the effect of DHA-PC from *P. crocea* roe on triglyceride accumulation in HepG2 cells.

## 2. Results and Discussion

### 2.1. Characterization of DHA-PC Liposomes

The liposomes prepared by the film dispersion method in this experiment were milky white suspensions. Liposomes were evenly distributed, showed a spherical shape, and the particles were dispersed well without aggregation. The average particle size of the liposomes was 234.4 nm, and the dispersion coefficient polydispersity index (PDI) was 0.209, indicating that the prepared liposomes had a uniform particle size distribution.

### 2.2. Cytotoxicity Evaluation

**Figure 1:**
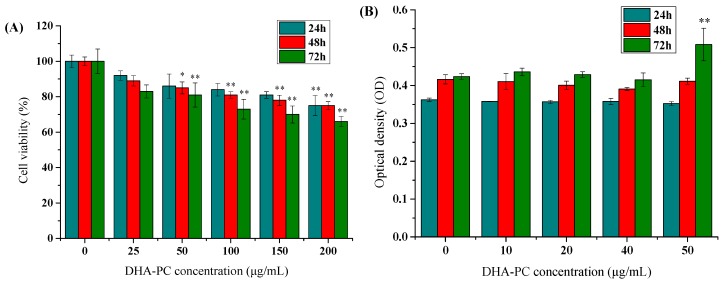
Cytotoxicity evaluation after treated with docosahexaenoic acid-containing phosphatidylcholine (DHA-PC) liposomes. (**A**) HepG2 cells were exposed to various concentrations (25, 50, 100, 150, 200 μg/mL) of DHA-PC liposomes for 24, 48, 72 h, and the cell viability was determined by MTT tests. The DHA-PC liposomes were considered not toxic to cells when the cell viability was more than 80%. On the basis of (**A**,**B**) HepG2 cells were exposed to various concentrations (10, 20, 40, 50 μg/mL) of DHA-PC liposomes for 24, 48, 72 h, and the release rate of lactate dehydrogenase (LDH) in HepG2 cells was expressed by the OD value. (**A**,**B**) The same volume of medium was added to the control (0 μg/mL) group. P-P plot was used to verify that the data met the normal distribution. ANOVA test was used for comparisons and Kolmogorov–Smirnov test was also used in Figure 1B. Compare with the control group, * *p* < 0.05, ** *p* < 0.01.

To determine whether different concentrations of DHA-PC liposomes were toxic to HepG2 cell, we used MTT calorimetry to evaluate the effect of sample concentration on cell viability. The results (Figure 1A) showed that, when treated for 24 h, there was no significant change in the proliferation activity of HepG2 cells until the treated concentration of DHA-PC liposomes reached 200 μg/mL, which produced an inhibition rate (IR) of 26%. With the prolongation of treatment time to 48 and 72 h, the inhibition effect enhanced. With the increase of the concentration of DHA-PC liposomes, the inhibition effect increased. The cell viability was still more than 80% when the concentration of DHA-PC liposomes was 50 μg/mL, indicating that DHA-PC liposomes was not toxic to the cells at the concentration of 50 μg/mL. The results were in agreement with those of Hwang et al. [34].

On the basis of these findings, the release of cellular lactate dehydrogenase (LDH) was also examined. The release of LDH from cells is an important indicator of cell membrane integrity, and hence was used to evaluate the cytotoxicity of DHA-PC liposomes. The release rate of lactate dehydrogenase (LDH) in HepG2 cells was expressed by optical density (OD) value. After treatment by 10, 20, 40, and 50 μg/mL of DHA-PC liposome for 24 and 48 h, the LDH activity of cells changed, but the differences were not significant. Compared with the control group, the content of LDH in the HepG2 cells treated with 50 μg/mL DHA-PC liposomes for 72 h was significantly higher than that of the control group, with a significant difference (*p* < 0.01). At the concentration of 50 μg/mL, DHA-PC liposomes showed a toxic effect on the cells (Figure 1B). Therefore, DHA-PC liposome concentrations 10, 25, and 40 μg/mL were selected for subsequent studies.

### 2.3. OA Induced HepG2 Cells to Establish a High-Fat Model in Vitro

**Figure 2:**
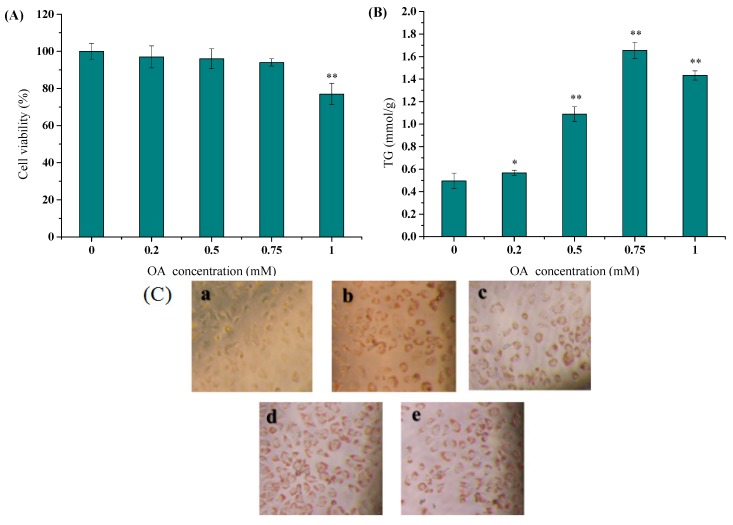
OA induced HepG2 cells to establish a high-fat model in vitro. (**A**–**C**) HepG2 cells were incubated with OA (oleic acid) at different concentrations (0.2, 0.5, 0.75, 1.0 mM). The cell viability was measured by MTT assay (**A**) and the intracellular TG content was determined according to the kit instruction (**B**). The OA was considered not toxic to cells when the cell viability was more than 80% (**A**). (**C**) OA-induced steatosis in HepG2 cells were determined by Oil Red O staining. P-P plot was used to verify that the results had no normality, then Kolmogorov–Smirnov test was used for comparisons. a: control; b: 0.2 mM OA; c: 0.5 mM OA; d: 0.75 mM OA; e: 1.0 mM OA. (**A**–**C**) The same volume of medium was added to the control (0 μg/mL) group. Compare with the control group, * *p* < 0.05, ** *p* < 0.01.

When the concentration was lower than 0.75 mM, OA had no effect on cell vitality (more than 80%). The cells began to die while the concentration of OA reached 1.0 mM (Figure 2A), indicating that OA had a certain inhibitory effect on cell viability at this concentration (*p < 0.01*). When the OA concentration was less than 0.75 mM, the triglyceride content increased as the OA concentration increased, reaching a maximum at 0.75 mM. As the OA concentration continued to increase to 1.0 mM, the TG content decreased (Figure 2B), and there was a significant difference (*p* < 0.01) when compared with control group (without adding OA). The HepG2 human hepatoma cells were selected as the cell line for the study because they retain many functions of normal hepatocytes, including secretion and metabolism of lipoproteins. In addition, the accumulation of free fatty acids in HepG2 cells can reach a large load, similar to human fatty liver [35]. Therefore, HepG2 cells were a good choice for establishing a high-fat model in vitro [36,37]. OA is a common fatty acid found in human plasma and is the final product of *de novo* synthesis of fatty acids. Excessive accumulation of OA can cause hepatic steatosis in humans [38]. OA is more steatogenic but less apoptotic than other saturated fatty acids (i.e., palmitic acid) in hepatocyte culture, indicating that OA has a protective effect on hepatocytes [39]. Chen et al. have demonstrated that OA has great potential to combat liver lipid toxicity induced by saturated fatty acids in hepatocytes and Nash rats [40].

There was no red lipid droplet deposition at the edge of HepG2 cells in the control group, while significant red lipid droplets were observed in the b, c, d and e groups. As the concentration of OA increased (0.2 to 0.75 mM), some lipid droplets became larger (Figure 2(Ca–d)). However, when treated with 1.0 mM OA, the number of cells was remarkably reduced; some cells were irregular in shape, and some motility was observed (Figure 2(Ce)). Therefore, when the concentration of OA selected was 0.75 mM, HepG2 cells showed no obvious proliferation defect. The cells contained a large number of lipid droplets, and the TG accumulation reached a high level (Figure 2(Cd)), indicating that the high-fat model in vitro was successfully established, which was basically consistent with previous reports [41,42]. However, there were differences in the concentration of OA used in different experiments.

### 2.4. Effect of DHA-PC on the Accumulation of Triglyceride in HepG2 Cells Induced by OA

**Figure 3:**
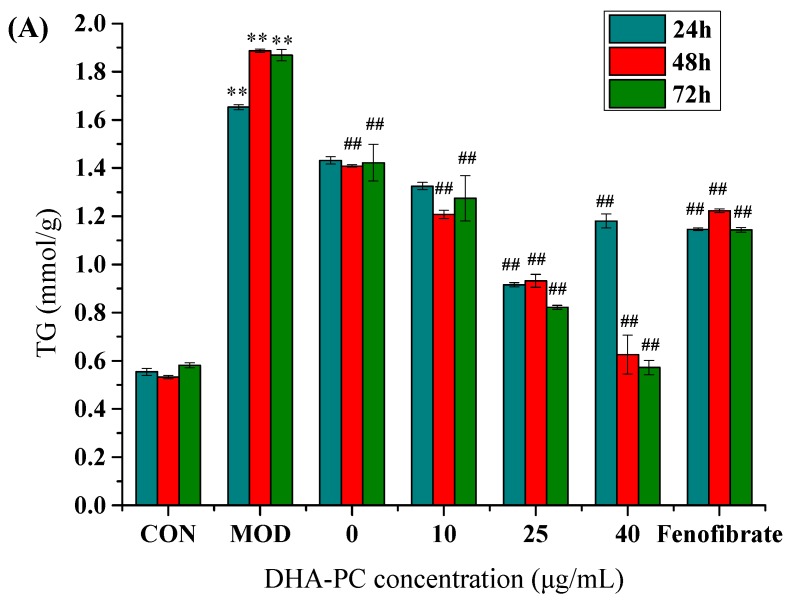

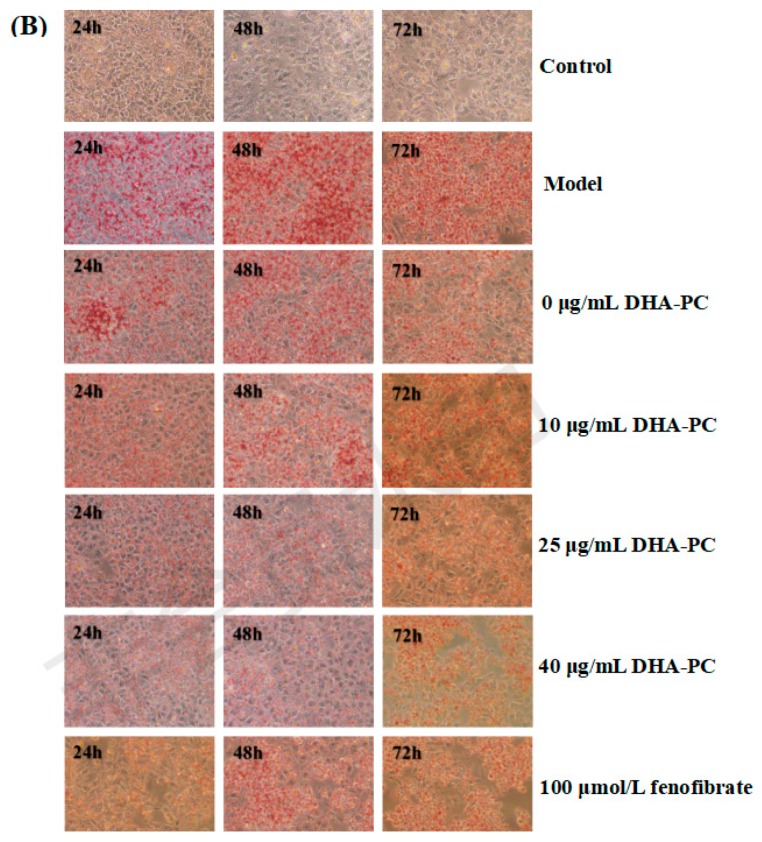
DHA-PC liposomes decreased the accumulation of triglyceride in HepG2 cells. (**A**,**B**) OA-induced HepG2 cells were exposed to various concentrations (0, 10, 25, 40 μg/mL) of DHA-PC liposomes for 24, 48, 72 h. 0 concentration group was regulated by the function of the cell itself. The same volume of medium was added to the control group, and the model group was added only with the same volume of 0.75 mM OA. The OA-induced HepG2 cells were treated with 100 μg/mL fenofibrate, a hypolipidemic drug. (**A**) The intracellular TG content was determined according to the kit instruction. P-P plot was used to verify that the results had no normality, then Kolmogorov–Smirnov test was used for comparisons. CON: control, MOD: model. Compared with the control group, ** *p* < 0.01; Compared with the model group, ^##^ *p* < 0.01. (**B**) OA-induced HepG2 cells were stained with oil red.

To quantitatively analyze the effect of DHA-PC on triglyceride (TG) deposition in HepG2 cells induced by OA, we determined the change of triglyceride content in the cells. The TG content of the model group (MOD) was significantly higher than that of the control group (CON) (*p* < 0.01), indicating successful modeling. Fenofibrate in the positive control group reduced intracellular lipid content, which was significantly different from the model group (*p* < 0.01). The untreated group was regulated by the function of the cell itself when compared with the model group, and the lipid content of this group was shown to decrease (Figure 3A). DHA-PC groups had better lipid-lowering effects, which decreased by 19.9%, 44.6%, and 28.6%, respectively when treated for 24 h.

Compared with 48 h, the lipid-lowering effect of DHA-PC was further improved after 72 h of treatment. After adding 40 μg/mL DHA-PC for 72 h, the lipid content of intracellular lipids decreased to 30.6% compared to the model group, and the lipid-lowering effect was better than that of the fenofibrate group. Therefore, 40 μg/mL was selected as the DHA-PC concentration for subsequent experiments. The results showed that prolonging the intervention time of DHA-PC could further enhance the lipid-lowering effect.

To observe the morphological effects of DHA-PC on OA-induced adipose accumulation in HepG2 cells, HepG2 cells were cultured for 24, 48, and 72 h with different concentrations of DHA-PC, stained with oil red O and observed under a microscope. The model group had clear cell edges with obvious red lipid droplets surrounding the cells, indicating successful model induction. As the concentration of DHA-PC increased, the red lipid droplets in HepG2 cells gradually decreased, the lipid droplets decreased, and the color became lighter. At the same concentration, the intracellular lipid droplets were further reduced with the extension of time (Figure 3B).

Tung et al. found that the supplementation comprising dietary fish oil with all-trans retinoic acid decreased blood lipids and fat accumulation in mice [43]. The results from Wang et al. showed that DHA-PC exhibited strong effects for improving the dysfunction of memory and cognition [44]. In our study, DHA-PC effectively reduced the deposition of lipid droplets in HepG2 cells, and the effect increased with the prolongation of intervention time. The lipid-lowering effect of DHA-PC group was better than that of fenofibrate, indicating that DHA-PC has a significant hypolipidemic effect. Thus, we propose that DHA-PC is a potential lipid-lowering drug.

### 2.5. Effect of DHA-PC on the mRNA Expression of Lipid Metabolism-Related Genes in HepG2 Cells

The above results showed that different concentrations of DHA-PC liposomes could reduce the accumulation of fat in HepG2 cell. However, the mechanism by which DHA-PC liposomes prevents the accumulation of fat in HepG2 cells was still not clear. Therefore, at the level of gene transcription level, we evaluated the effect of exposure to 40 μg/mL DHA-PC liposomes on the expression of FAS, CPT1A and PPARα in HepG2 cells by RT-qPCR.

#### 2.5.1. mRNA Expression of Lipid Metabolism-Related Genes (FAS) in HepG2 Cells

**Figure 4:**
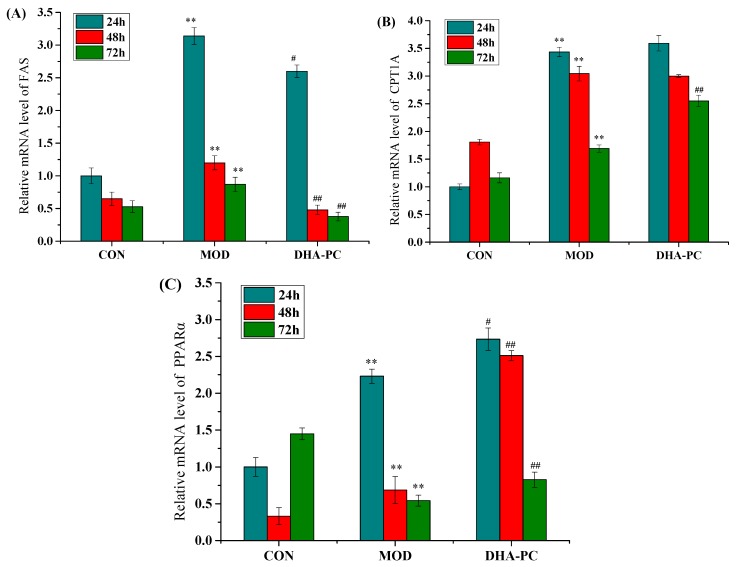
DHA-PC down-regulated the mRNA expression of FAS and up-regulated mRNA expressions of CPT1A and PPARα in HepG2 cells. (**A**–**C**) OA-induced HepG2 cells were exposed to DHA-PC liposomes of 40 μg/mL for 24, 48, 72 h. The same volume of medium was added to the control group, and the model group was added only with the same volume of 0.75 mM OA. The mRNA expressions of FAS (**A**), CPT1A (**B**) and PPARα (**C**) in HepG2 cells were detected by RT-qPCR. Expression levels were normalized to that of GAPDH mRNA expression. P-P plot was used to verify whether the data met the normal distribution. ANOVA test was used for comparisons in Figure 4B,C. Kolmogorov–Smirnov test was also used in Figure 4A. CON: control, MOD: model. Compared with the control group, ** *p* < 0.01; Compared with the model group, ^#^ *p* < 0.05, ^##^ *p* < 0.01.

The expression of FAS mRNA in the model group increased compared with the control group, and the difference was statistically significant (*p* < 0.01). Compared with the model group, the DHA-PC liposomes effectively reduced the expression of FAS mRNA in HepG2 cells induced by OA (Figure 4A), and the difference was statistically significant (*p* < 0.05). Among them, the effect of DHA-PC liposome on the expression of FAS mRNA in HepG2 cells was the most significant after 72 h, which was 56.4% lower than that of model group under the same treatment time. The results showed that the expression of FAS mRNA in HepG2 cells decreased with the prolongation of time, revealing a certain time-dependent effect. FAS is a key enzyme in the synthesis of fatty acids [45]. It is a multifunctional complex enzyme with multiple regulatory sites, effectively inhibiting the expression of FAS and the synthesis of fatty acids. It has been proven that the effect of n-3 PUFAs-containing PC (n-3-PC) on reducing lipid accumulation may be related to enzyme activity (such as CPT) and, specifically, to the reduction of fat synthase activity (such as FAS), which is related to the increase of fat metabolism [46].

#### 2.5.2. mRNA Expression of Lipid Metabolism-Related Genes (CPT1A) in HepG2 Cells

Compared with the control group, the expression of CPT1A mRNA in the model group increased (Figure 4B), and the difference was statistically significant (*p* < 0.01). There was no significant difference in the expression of CPT1A mRNA in the HepG2 cells after DHA-PC treatment for 24 and 48 h. The expression of CPT1A mRNA in DHA-PC cells treated for 72 h was significantly higher than that of the model group, producing an increase in expression of 1.5 times that of the model group, which was found to be a significant difference (*p* < 0.01). CPT1A has a widespread distribution and is found in liver, pancreas, kidney, brain, blood and embryonic tissues [47,48]. Liu et al. also found that the lipid-lowering effect on HepG2 cells was mediated by the upregulation of CPT1 and FAS gene expression [49].

#### 2.5.3. mRNA Expression of Lipid Metabolism-Related Genes (PPARα) in HepG2 Cells

Compared with the control group, the expression of PPARα mRNA in the model group increased when the model groups were treated with DHA-PC for 24 and 48 h (Figure 4C). The difference was statistically significant (*p* < 0.01). DHA-PC was added to another model group for 72 h; compared with the control group, the expression of PPARα mRNA in the model group decreased, and there was a significant difference (*p* < 0.01). Compared with the model group, the expression of PPARα mRNA in the DHA-PC liposome group was significantly increased in HepG2 cells. Among these groups, the effect on the expression of PPARα mRNA in HepG2 cells treated with DHA-PC for 48 h was the most obvious; the expression of PPARα was 3.6 times greater than that of the model group in the same period, and the difference was statistically significant (*p* < 0.01). PPARα is an important subtype of the PPARs family [50]. It is a nuclear hormone receptor that regulates the expression of genes involved in lipid metabolism and is expressed in many cell types, such as T lymphocytes, macrophages, liver and kidney cells [51,52].

### 2.6. Effect of DHA-PC on the Expression of Lipid Metabolism-Related Proteins in HepG2 Cells

**Figure 5:**
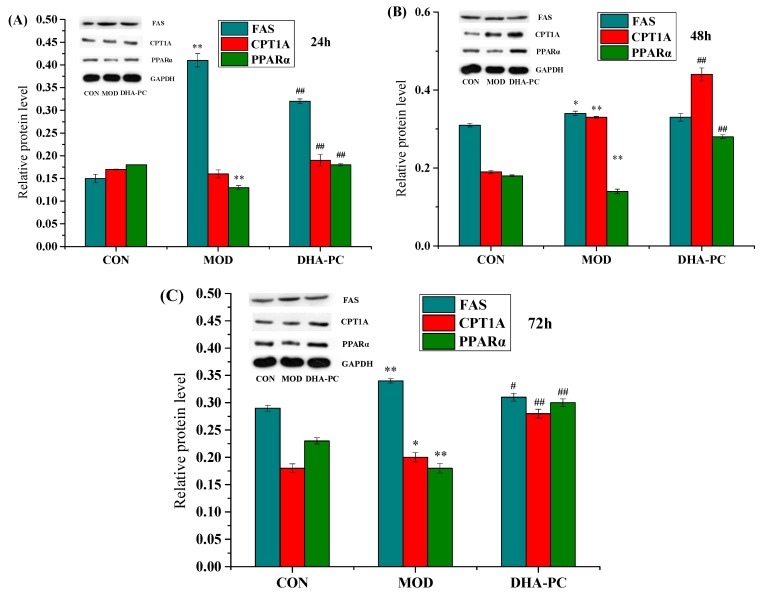
DHA-PC down-regulated the protein expression of FAS and up-regulated protein expressions of CPT1A and PPARα in HepG2 cells. OA-induced HepG2 cells were exposed to DHA-PC liposomes of 40 μg/mL for 24 (**A**), 48 (**B**), 72 h (**C**). (**A**–**C**) The same volume of medium was added to the control group, and the model group was added only with the same volume of 0.75 mM OA. The protein expressions of FAS, CPT1A and PPARα and the gray value corresponding to each protein expression in HepG2 cells were detected by Western blot. Expression levels were normalized to that of GAPDH protein expression. P-P plot was used to verify that the data met the normal distribution. ANOVA test was used for comparisons. CON: control, MOD: model. Compared with the control group, * *p* < 0.05, ** *p* < 0.01; Compared with the model group, ^#^ *p* < 0.05, ^##^ *p* < 0.01.

After 24, 48, and 72 h, FAS protein expression in the model group was higher than that in the control group, and the expression of PPARα protein in the model group was lower than that in the control group (Figure 5), with significant differences (*p* < 0.01). When the cells were treated for 24 h, the expression of CPT1A protein in the model group was not significantly different from that in the control group (Figure 5A). The trend of CPT1A protein expression in the model group was consistent with that of FAS after 48 and 72 h, and the difference was statistically significant (*p* < 0.01). Compared with the model group, DHA-PC had a downregulation effect on the protein expression of FAS, and the difference was significant (*p* < 0.01) after 24 and 72 h. However, there was no significant difference when the cells were treated for 48 h (Figure 5B). The expression of both CPT1A and PPARα protein was upregulated after treatment of 24, 48 and 72 h, with a significant difference (*p* < 0.01).

It has been revealed that elevated FAS expression can increase the accumulation of TG in the body, leading to obesity [53]. This study found that, compared with the model group, the DHA-PC liposome group showed reduced expression of FAS mRNA and protein in HepG2 cells at different time points. However, there was no significant difference in the expression of CPT1A mRNA in HepG2 cells treated with DHA-PC liposomes for 24 and 48 h. The expression of CPT1A mRNA increased significantly in the DHA-PC group when the treatment time was prolonged to 72 h. Therefore, the relationship between CPT1A and fatty acid metabolism needs further confirmation. Moreover, we also found that DHA-PC can increase the expression of PPARα mRNA and protein in HepG2 cells when compared with the control. It has been reported that activation of PPARα receptor activity is effective in reducing TG levels in hepatocytes [54,55,56]. However, we only identified a limited number of genes (FAS, CPT1A and PPARα) involved in lipid metabolism in HepG2 cells. Thus, further studies should be performed to confirm our findings.

The above results showed the regulatory effect of hyperlipidemia mediated by DHA-PC from *P. crocea* roe may be related to the downregulation of FAS, upregulation of CPT1A and activation of PPARα. These results laid a theoretical foundation for further study on the molecular mechanism by which DHA-PC regulates lipid metabolism in HepG2 cells. More work is still needed to elucidate the specific lipid metabolic pathways.

## 3. Materials and Methods

### 3.1. Chemicals and Reagents

Reagents and medium were purchased from Sigma (St. Louis, MO, USA), unless otherwise noted. DMEM high sugar medium and fetal bovine serum were purchased from Gibco (Grand Island, NY, USA). Streptomycin solution, trypsin-EDTA digestive cocktail (0.25%), dimethyl sulfoxide (DMSO), 4% paraformaldehyde, Oil red O dye and cell lysis buffer were obtained from Solaibao Technology Co., LTD (Beijing, China). MTT kit and LDH kit were purchased from Keji Biotechnology Co., LTD (Nanjing, China). TG kit was purchased from Nanjing Jiancheng Biological Engineering LTD (Nanjing, China). The BCA protein quantitation kit was obtained from Jinfu Sai Biotechnology Co., LTD (Beijing, China). Cholesterol and chloroform were purchased from National drug chemical reagents Co., LTD (Shanghai, China). Agarose was purchased from Biowest (Barcelona, Spain). Trizol, SYBR Green PCR Mix and RT-qPCR kits were obtained from Invitrogen (Carlsbad, CA, USA).

### 3.2. Preparation of DHA-PC Liposomes

The DHA-PC was isolated and purified from *P. crocea* roe in our previous work [57]. Total cholesterol (TC) and DHA-PC with a mass ratio of 1:1.5 were dissolved in a round bottom flask with a volume of 500 mL with a small amount of chloroform, and rotary evaporation was carried out at a temperature of 30 °C to obtain a dry phospholipid film. A certain amount of D-Hanks was added and mixed, then processed on the ultrasound system. DHA-PC liposomes were obtained after 200 nm microporous filter membrane filtration.

### 3.3. Characterization of DHA-PC Liposomes

The DHA-PC liposomes were prepared in suspension in double-distilled water. After 2 min, the liposomes were dyed with 2% phosphotungstate for 1 min; then, the liposomes were dried naturally and observed with a scanning electron microscope [58]. The prepared DHA-PC liposomes were placed in a nanometer particle size analyzer to determine their particle size. Each sample was generated in triplicate.

### 3.4. Cell Culture and Treatment

The HepG2 cells purchased from the Nanjing KGI Biotechnology Co., LTD (Nanjing, China) were cultured in DMEM complete medium with high glucose and 10% (vol/vol) fetal bovine serum at 37 °C. The incubator contained 5% CO_2_, and the humidity was 100%.

When the cells were over 80% confluent, it was necessary to carry out the passage of HepG2 cells. 1 mL 0.25% trypsin (containing 0.02% EDTA) was added to the bottle for digestion. The trypsin was discarded when the cells shrank and became round. The digestion was terminated by adding DMEM complete medium, and the cells were gently pipetted to separate them from the bottle wall. The cell suspension was aspirated and dispensed into a culture flask to continue the culture. The cells with good growth state were frozen, and the frozen cells were resuscitated and transferred to a culture flask for routine culture prior to use in subsequent experiments.

### 3.5. Cytotoxicity Evaluation

#### 3.5.1. Determination of HepG2 Cell Viability Affected by Different DHA-PC Liposome Concentrations

HepG2 cells were digested with 0.25% trypsin, and cell suspension with a concentration of 2 × 10^5^ cells/mL was prepared in DMEM complete medium; then, the suspension was cultured in a 96-well culture plate at 100 μL/well for 24 h. In the control group, only culture medium was added, and the experimental group was treated with DHA-PC liposomes at concentrations of 25, 50, 100, 150, and 200 μg/mL. Five replicate wells were set in each group, and the plate was placed in a cell culture incubator (37 °C, 5% CO_2_) for 24, 48, and 72 h. Four hours before the termination, 20 μL MTT solution was added to each well; then, the culture was continued for another 4 h. Subsequently, 150 μL DMSO was added per well. An oscillator was used for 5-10 min to completely dissolve the blue-purple crystals. The OD value at 490 nm was measured by the microplate reader, and the cell survival rate was represented by T (sample group OD value)/ C (control group OD value).

#### 3.5.2. Determination of Lactate Dehydrogenase

HepG2 cells were inoculated at a cell concentration of 1 × 10^5^ cells/mL in 96-well plates at 100 μL/well. After culturing for 24 h, the culture solution was discarded. The same volume of medium was added to the control group, and the experimental group was treated with DHA-PC liposomes at concentrations of 10, 20, 40, and 50 μg/mL. Five replicate wells were set in each group, and the plate was placed in a cell culture incubator (37 °C, 5% CO_2_) for 24, 48, and 72 h. After the treatment, the cell culture supernatant was collected, and the release rate of LDH was detected according to the instructions of the LDH detection kit.

### 3.6. OA Induced HepG2 Cells to Establish a High-Fat Model in Vitro

MTT, TG, and Oil red O staining experiments were carried out on cells from the model cell line. The optimum concentration of OA was selected according to the cell viability, TG content, and dyeing effect [59].

#### 3.6.1. Determination of HepG2 Cell Viability Induced by Different Concentrations of OA

HepG2 cells passaged to the fourth passage were inoculated at a cell concentration of 2 × 10^5^ cells/mL in a 96-well plate at 100 μL/well. After the cells adhered to the wall, the culture medium was discarded, and the control group and the model group were prepared. Five replicate wells were set in each group. The control group was cultured in DMEM complete medium, and the model group was cultured for 24 h with medium containing different concentrations of OA (0.2, 0.5, 0.75, and 1.0 mmol/L). The viability of the cells was measured by MTT assay as described in 3.5.1.

#### 3.6.2. Determination of TG content in HepG2 Cells

After adding trypsin for digestion, the medium was added to terminate the digestion. The medium was gently pipetted several times, and the cell suspension was collected. Next, the cell pellet was collected after cells were centrifuged at 1000 rpm/min for 5–10 min. Then, the pellet was washed twice with PBS, and the cells added with cell lysis buffer were lysed on ice for 30 min, which was followed by centrifugation at 10,000 rpm/min for 10 min at 4 °C; then, the supernatant was collected. The TG content was then determined according to the kit’s instructions, by the single reagent GPO-PAP method according to Nanjing Jiancheng Biological Engineering LTD.

#### 3.6.3. Oil Red O Staining of Intracellular Lipids

After treatment, 4% paraformaldehyde was added to cells for 15–30 min, and cells were washed twice with PBS and dyed with 60% isopropanol for 1–2 min. Then, 1.5 mL of Oil Red O staining solution was added to the culture plate, and the mixture was kept in the dark for 0.5–1 h. The dye solution was carefully removed from the edge; the slide was rinsed twice with distilled water and observed and imaged with an inverted microscope. Each sample was generated in quintuplicate.

### 3.7. Detection of mRNA Expression of HepG2 Cell-Related Genes by RT-qPCR

Total RNA was isolated from the HepG2 cells using TRIzol reagent. The purity of RNA was calculated by the following formula: OD_260_ × diluent factor × 40. The extracted total RNA was used as a template for reverse transcription of cDNA. Reverse transcription product cDNA was subjected to RT-PCR amplification using the Access RT-PCR System. The following primers were used, and the size of the amplicon is given in parentheses: FAS, forward-CGTCTGTTGCTAGATTATCGTCC and reverse-CTGTGCAGTCCCTAGCTTT (186 bp); CPT1A, forward-TCCAGTTGGCTTATCGTGGTG and reverse-TCCAGAGTCCGATTGATTTTTGC (98 bp); PPARα, forward-AAACAAATGCCAGTATTGTCGATT and reverse-CTTTCTCAGATCTTGGCATTCGTC (99 bp); GAPDH, forward-ACAGCCTCAAGATCATCAGC and reverse-GGTCATGAGTCCTTCCACGAT (104 bp). The amplification conditions, such as the number of amplification cycles, were determined according to a kinetic profile. GAPDH was used as an internal control. Ten microliters of RT-qPCR product from each group of cells was mixed with DNA Marker and was subjected to gel electrophoresis in 1.5% agarose, observed and photographed. Gel-Pro software was used to analyze the grayscale ratio of electrophoresis bands. All RT-qPCR reactions were normalized against GAPDH expression. Three replicate samples for each test.

### 3.8. Detection of Protein Expression of HepG2 Cell-Related Genes by Western Blot

After treatment, HepG2 cells were washed 3 times with cold PBS and lysed with RIPA lysis buffer. The assay was performed according to the instructions of the BCA kit, and the protein concentration was calculated according to the standard curve. The same amount of protein for each sample was mixed with loading buffer, boiled for 5 min and quickly cooled prior to loading for SDS-PAGE. Proteins were blotted onto nitrocellulose filter (NC) membranes, which were then blocked by 5% skimmed milk formulated with TBST for 1.5 h at room temperature. The NC membranes were then incubated with specific primary antibodies overnight at 4 °C, followed by washing 3 times with TBST for 15 min each time. Subsequently, HRP-labeled secondary antibodies were incubated for 1.5 h at room temperature and then washed 3 times with TBST for 15 min each time. Finally, the blots were visualized using an enhanced assay kit, and the bands were quantified using image software. Three parallel samples for each test.

### 3.9. Statistical Analysis

The experimental data were expressed as the mean ± standard error of the mean (SEM). All data were subjected to analysis of variance using SPSS 13.0 software. The P-P plot was used to verify that the data met the normal distribution before using one-way ANOVA for comparisons. The differences among control, OA, fenofibrate and DHA-PC groups were analyzed by one-way ANOVA followed by the Student’s t-test. If there were no normality in the results, then non-parametric comparison (Kolmogorov–Smirnov test) was used instead. And *p* < 0.05 was considered to be statistically significant.

## 4. Conclusions

DHA-PC could significantly regulate the accumulation of triglyceride in HepG2 cells, which was evidenced by the reduced level of intracellular triglycerides. We speculated that the biofunction utilized by DHA-PC to lower blood lipids may be related to the activation of PPARα receptor activity, upregulation of CPT1A, and downregulation of FAS expression. In this study, it was concluded that DHA-PC can exert a positive effect on the regulation of hyperlipidemia by DHA-PC from *P. crocea* roe, contributing to the development of DHA-PC of *P. crocea* roe as a functional food additive and the improvement of human health. Further works are still necessary to illuminate the specific molecular mechanism which DHA-PC from *P. crocea* roe exerts the hyperlipidemic modulatory effects.

## Abbreviations

PLs	phospholipids
*P. Crocea*	large yellow croaker
DHA	docosahexaenoic acid
DHA-PC	DHA-containing phosphatidylcholine
OA	oleic acid
FAS	fatty acid synthase
CPT1A	carnitine palmitoyl transferase 1A
PPARα	peroxisome proliferator-activated receptor α
PC	phosphatidylcholine
n-3 PUFAs	n-3 polyunsaturated fatty acids
EPA	eico-sapentaenoic acid
PUFAs	polyunsaturated fatty acids
PAF	Platelet-activating factor
PL	phospholipid
DHA-PL	DHA-containing phospholipids
PE	phosphatidylethanolamine
PDI	polydispersity index
IR	inhibition rate
LDH	lactate dehydrogenase
OD	optical density
TG	triglyceride
n-3-PC	n-3 PUFAs-containing PC
TC	total cholesterol
NC	nitrocellulose filter
